# P-2147. Incidence of Invasive Fungal Infection in Venetoclax-based Regimens for AML/MDS Patients on Micafungin Prophylaxis

**DOI:** 10.1093/ofid/ofae631.2301

**Published:** 2025-01-29

**Authors:** Williams Monier Texidor, Lee Amaya, Timothy Gauthier, Marco Ruiz Andia

**Affiliations:** Miami Cancer Institute Baptist Health South Florida, Miami, Florida; Miami Cancer Institute Baptist Health, Miami, Florida; Baptist Health South Florida, Miami, Florida; Miami Cancer Institute Baptist Health, Miami, Florida

## Abstract

**Background:**

Combining venetoclax with a hypomethylating agent (HMA) is standard of care in Acute Myeloid Leukemia (AML) ineligible for intensive chemotherapy. However, this agent is associated with prolonged neutropenia, potentially increasing the risk of invasive fungal infections (IFI). The necessity of antifungal prophylaxis in this context remains unclear, particularly in regions like the southeastern United States, where the humid climate may contribute to higher overall rates of IFIs.

**Figure:**
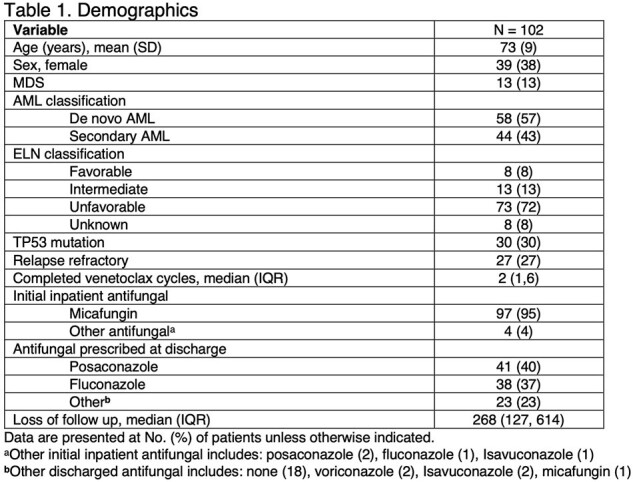

**Methods:**

A retrospective, observational cohort study evaluated patients admitted to Baptist Health Baptist Hospital in Miami, Florida, from January 2019 to January 2023 who received venetoclax and an HMA with/without low-dose cytarabine for AML/MDS. Exclusions included incomplete first cycle, active IFI treatment, absence of antifungal prophylaxis, or initiation of venetoclax at an outside institution. The primary endpoint was 90-day IFI incidence using 2022 EORTC criteria. The secondary endpoints were complete response (CR) per ELN 2022 guidelines, 1-year mortality, and 90-day count recovery.

**Figure:**
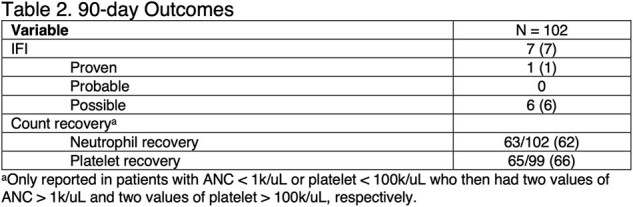

**Results:**

Among 102 patients evaluated, the mean±SD age was 73±9 years. Twenty-seven patients (27%) had relapsed refractory disease, and 42 (41%) received prior chemotherapy. Overall, 57% presented with de novo AML/MDS; 13 treated specifically for MDS. Micafungin was the predominant inpatient prophylaxis agent (95%), while posaconazole (40%) and fluconazole (37%) were the predominant prophylaxis agents prescribed at discharge. IFI incidence at 90 days was 7%, consisting mostly of possible IFI (6/7). CR was achieved in 61% of patients, and 1-year mortality was 32%. 90-day neutrophil recovery occurred in 62% (63/102), while platelet recovery occurred in 66% (65/99).

**Figure:**
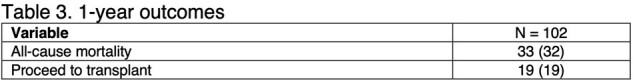

**Conclusion:**

Our findings suggest that micafungin as primary antifungal prophylaxis in AML/MDS patients receiving venetoclax-based regimens demonstrates a comparable incidence of IFI at 90 days to previous studies reporting similar outcomes.

**Disclosures:**

Timothy Gauthier, PharmD, BCPS, BCIDP, AbbVie Pharma: Advisor/Consultant|Antimicrobial Therapy, Inc: Advisor/Consultant|Ferring Pharma: Advisor/Consultant|Firstline Mobile Health: Advisor/Consultant|Gilead Pharma: Advisor/Consultant|GoodRx: Advisor/Consultant|GSK Pharma: Advisor/Consultant|Melinta Pharma: Advisor/Consultant|Pattern Biosciences: Advisor/Consultant|Pfizer Pharma: Advisor/Consultant|ProCE: Honoraria|WWW.LearnAntibiotics.com: Ownership Interest

